# Seroprevalence of Anti-SARS-CoV-2 Antibodies Following the Omicron BA.1 Wave

**DOI:** 10.3390/ijerph20043665

**Published:** 2023-02-18

**Authors:** Maja Socan, Katarina Prosenc, Maja Mrzel

**Affiliations:** 1 National Institute of Public Health, 1000 Ljubljana, Slovenia; 2 National Laboratory for Health, Food and Environment, 1000 Ljubljana, Slovenia

**Keywords:** seroprevalence, Omicron BA.1, COVID-19, anti-nucleocapsid protein antibodies, anti-spike glycoprotein antibodies

## Abstract

We conducted a seroprevalence study using convenient residual sera samples from the Slovenian population collected after the end of the Omicron BA.1 pandemic wave. Serum samples were tested for spike glycoprotein (anti-S) and nucleocapsid protein (anti-N) antibodies. Participants’ data regarding confirmed infection and vaccination was obtained from national registries. Anti-S antibodies were detected in 2439 (84.1%) of 2899 sera from persons aged 0–90 years, with the lowest prevalence in the 0–17 age group. The proportion of anti-N positives was the lowest in the ≥70 age group. The proportion of anti-N positives was significantly higher among participants with confirmed past infection and among those who had never been vaccinated. In participants who had not been notified as infected and who had never been vaccinated, the seroprevalence of anti-S and anti-N antibodies was 53% and 35.5%, respectively. From the time of serum collection to mid-November 2022, 445 participants (15.3%) tested positive for SARS-CoV-2, with higher odds in seronegative participants, participants in the 40–59 age group, and those without notified previous infection. Vaccination status and gender had no significant effects on infection risk. This study underlines the importance of serosurveys in understanding the development of the pandemic.

## 1. Introduction

Infection with the SARS-CoV-2 virus elicits a humoral and cell-mediated immune response [1]. Humoral immune responses to the SARS-CoV-2 virus are mediated by antibodies that are directed to viral surface glycoproteins, predominantly to the spike (S) glycoprotein and the nucleocapsid (N) proteins, which are the main immunogens. The S protein is a major protective antigen that provokes highly potent neutralising antibodies and plays a crucial role in viral attachment, the fusion of viral and host membranes, and the entry of the virus into host cells [2,3]. Antibodies neutralise the viral infection of human cells and tissues expressing angiotensin-converting enzyme 2 (ACE2) [4]. Most individuals infected with SARS-CoV-2 develop antibodies to the S and N proteins, which are therefore used as antigens in clinical serology assays. The kinetics of the antibody response against SARS-CoV-2 is characterised by seroconversion 1–2 weeks following symptom onset, with detectable antibody concentrations persisting for several months after infection [4]. The dynamics of the humoral immune response during the acute phase of COVID-19 and soon afterwards have been well documented and studied [3,5,6].

Serological studies showed that the magnitude of serological immune responses is highly variable [7]. Some early and more recent studies suggested that patients with more severe illnesses eventually had higher antibody titres than those with a milder form of the disease [8,9]. Not everyone infected with the SARS-CoV-2 virus was seroconverted and asymptomatic or oligosymptomatic and/or had higher minimum cycle thresholds. RT-PCR was the most consistent factor in non-seroconversion found in prospective cohort studies [10,11]. Studies also revealed that immunocompromised patients had a consistently lower prevalence of anti-SARS-CoV-2 antibodies compared to immuno-competent persons [12]. Liver transplant recipients showed a lower prevalence of anti-nucleocapsid and anti-spike IgG antibodies one year after SARS-CoV-2 infection [13]. Kidney transplant patients were able to generate normal (but delayed) serum levels of anti-SARS-CoV-2 IgG upon infection, with serum antibody levels decreasing more rapidly compared with immunocompetent subjects [14].

The long-lasting durability (>12 months) of the antibody response has been described in longitudinal studies, which showed that the seropositivity declined to both anti-S and anti-N proteins, with a higher stability of antibodies directed to the anti-S protein, while anti-N antibodies were less well maintained [15,16,17]. The results of these studies suggest that a durable humoral immune response reduces the risk of reinfection, or at least of severe reinfection, within, as a minimum, one year [16].

The aim of our study was to determine the seroprevalence of anti-N and anti-S antibodies in a convenience sample of the Slovenian population after the end of the Omicron BA.1 pandemic wave and to correlate the results with prior SARS-CoV-2 infection and vaccination. We also compared the frequency of post-sampling infection in seropositive and seronegative individuals.

## 2. Participants and Methods

### 2.1. Study Participants

For this cross-sectional, age-stratified seroprevalence study, a total of 2899 sera from persons aged 0–90 years were tested for anti-N and anti-S SARS-CoV-2 antibodies. The serum samples were convenient, non-random samples obtained from residual sera collected during routine laboratory testing at the National Laboratory of Health, Environment and Food (NLHEF). Serum samples were gathered between 1 March and 6 June 2022. The sera were derived from all age groups, with an uneven gender distribution (males 672, 23.2% and females 2227, 76.8%, Supplementary Material: Table S1. Sample age and gender distribution).

### 2.2. Serologic Testing

Blood was collected using the standard venipuncture technique. Whole blood was centrifuged, and the serum was separated and used for primary testing. The sera were then anonymised and stored in a cryotube at −20 °C until testing for SARS-CoV-2 for the purposes of this study. We used the EUROIMMUN SARS-CoV-2 ELISA (IgG) test to determine the presence of SARS-CoV-2 IgG antibodies against the S protein and the Anti-SARS-CoV-2 NCP ELISA (IgG) test to determine the presence of IgG antibodies against N protein (both EUROIMMUN, Lübeck, Germany). The tests use microtiter plate wells coated with the S1 domain of the spike protein as an antigen and with the modified nucleocapsid protein (NCP), respectively. The tests were performed in accordance with the manufacturer’s recommendations. In summary, the sera were diluted 1:101 in a sample buffer and incubated at 37° for 60 min in a 96-well microtiter plate. This was followed by washing. Conjugate (peroxidase-labelled anti-human IgG) was added and incubated at 37° for 30 min for IgG against the S protein and at room temperature for 30 min for IgG against the N protein. This was again followed by washing. The plate with the substrate was incubated at room temperature for 30 min for IgG against the S protein and for 15 min for IgG against the N protein. Stop solution was added and the optical density (OD) was measured at 450 nm (microplate reader Sunrise, Tecan, Grödig, Austria). The prescribed controls and calibrator were used. All study sera were tested using the same batch of kits. The results were evaluated semi-quantitatively by calculating the ratio of the extinction of the control or patient sample over the extinction of the calibrator. The ratio values were calculated, and the results were interpreted in accordance with the manufacturer’s protocol (<0.8 negative; ≥0.8 to <1.0 borderline; ≥1.1 positive). Sera that gave inconclusive (borderline) results were tested again in duplicate, and the evaluation was resolved. The inter-assay coefficient of variability was calculated from positive controls that were used on each plate, and this was 4.2% for IgG against the S protein and 3.5% for IgG against the N protein. The intra-assay coefficient was calculated from specimens that were run in duplicates. It was 1.5% and 1.4% for IgG against the S and N proteins, respectively.

Before the study sera were tested, ELISA assays from different producers were studied for their sensitivity and specificity in the literature [18,19]. Four days after the positive PCR test, the reported sensitivity was already 100%, and the specificity was 97.7%. In addition, EUROIMMUN SARS-CoV-2 IgG ELISA was also tested and validated in-house on 72 sera from patients with a known SARS-CoV-2 infection history. The sensitivity was 100% for sera that were taken 14 or more days after the onset of illness. One sample each of the sera that tested positive for hCoV 229E, hCoV HKU1, hCoV NL63, hCoV OC43, adenovirus, rhinovirus, influenza A, and hMPV, were all negative for SARS-CoV-2 IgG, and all sera from asymptomatic PCR negative individuals were negative (data not published).

### 2.3. Data Management and Statistics

#### 2.3.1. Sources of Data

Two national health administration databases containing individual health information data were used: the National COVID-19 Database and the National Vaccination Register.

The National COVID-19 Database is a part of the National Notifiable Communicable Diseases Database. The database covers all laboratory-confirmed SARS-CoV-2 cases (symptomatic and asymptomatic) in Slovenia. SARS-CoV-2 infection was confirmed by RT-PCR tests or rapid antigen detection tests (RAT). The National COVID-19 Database is linked to the Central Registry of Patient Data and some other health administration data not relevant to the current study. The data extracted from the National COVID-19 Database included age (in years), gender, and dates of confirmed infections (primary infections and reinfections). The time interval between serum collection and SARS-CoV-2 infections (in days) was calculated. We extracted data from the National COVID-19 Database on SARS-CoV-2 infections from the beginning of the pandemic in Slovenia (4 March 2020) to 14 November 2022. We, therefore, recorded all infections before and after serum sampling and until the above-mentioned date.

We obtained data on vaccination against COVID-19 from the eRCO National Vaccination Register (Slovenian: Elektronski register cepljenih oseb, ‘Electronic Register of Vaccinated Persons’). The data extracted from the eRCO register was the date of the vaccination and the vaccine used.

#### 2.3.2. Definitions

For the classification of study participants, we used the following definitions:

1. Not infected with SARS-CoV-2 before serum sampling: participants without notified infection (no record in the National COVID-19 Database before serum sample collection or in the 14 days afterwards).
2. Non-vaccinated: participants who did not have any anti-SARS-CoV-2 vaccine dose recorded before the date of serum sample collection, or had only one dose of a two-dose schedule vaccine (the mRNA vaccines Comirnaty [Pfizer/BioNTech] or Spikevax [Moderna], vector vaccine Vaxzevria [Astra-Zeneca] or protein vaccine Nuvaxovid (Novavax]), or one dose of Jcovden/Janssen (Johnson & Johnson, New Brunswick, NJ, USA) in the 14-day period before the serum sample was taken.
3. Partially vaccinated: participants who had received only one dose of a two-dose schedule vaccine (mRNA vaccines Comirnaty [Pfizer/BioNTech] or Spikevax [Moderna], vector vaccine Vaxzevria [Astra-Zeneca] or protein vaccine Nuvaxovid [Novavax]) at least 14 days before serum collection.
4. Completely vaccinated: participants who received one dose of the Jcovden/Janssen (Johnson & Johnson) vaccine or both doses of a two-dose schedule vaccine (mRNA vaccines Comirnaty [Pfizer/BioNTech] or Spikevax [Moderna], vector vaccine Vaxzevria [Astra-Zeneca] or protein vaccine Nuvaxovid [Novavax]) at least 14 days before serum collection.
5. Vaccinated with additional dose: completely vaccinated participants who received at least one additional dose of an mRNA-based vaccine (Comirnaty [Pfizer/BioNTech] or Spikevax [Moderna]) before serum sampling.

For analytical purposes, we further classified the participants into four categories according to the data in the National COVID-19 Database and National Vaccination Register:

– persons who were not infected with SARS-CoV-2 and had not been vaccinated before sampling (never infected-never vaccinated);
– persons with at least one notified infection at least 14 days before serum sample collection AND never vaccinated (infected-never vaccinated);
– persons not infected with SARS-CoV-2 before sampling AND partially or completely vaccinated, or who had received at least one additional dose (never infected-vaccinated);
– persons with at least one notified infection at least 14 days before serum sample collection AND partially or completely vaccinated, or who had received at least one additional dose (infected-vaccinated).

#### 2.3.3. Statistical Analysis

First, the overall seroprevalence of anti-S and anti-N antibodies was examined. To calculate the crude and adjusted odds ratios and 95% confidence intervals, logistic regression was used. Other variables included in the model were gender, age, previous infections, and vaccination status. Second, the participants were stratified and seroprevalence was compared according to previous infections and vaccination status, and in relation to the number of months since the last recorded event (vaccination or infection). In this study, one month was defined as a 30-day period.

Finally, the risk of infection was evaluated in relation to anti-S and anti-N antibodies, gender, and age, while previous infection and vaccination status were eliminated from the multiple logistic regression due to the strong correlation with seropositivity.

SPSS statistical software (version 27, IBM, Armonk, NY, USA) was used to perform data analysis.

## 3. Results

### 3.1. COVID-19 Pandemic Development and Predominance of the Variants

**Figure 1:**
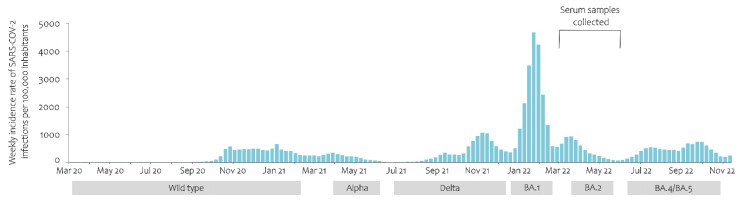
SARS-CoV-2 infection incidence rate (per 100,000) and variant predominance in Slovenia from the first detected case (4 March 2020) to 14 November 2022.

Figure 1 shows the evolution of the pandemic in Slovenia, the predominance of SARS-CoV-2 variants, and the timing of the collection of serum samples for the seroprevalence study. The SARS-CoV-2 variant was designated as predominant when ≥75% of sequenced viruses belonged to the same variant subgroup. For variant screening, the random selection of at least 10% RT-PCR positive specimens was sequenced. Sequencing was also performed for specimens of special interest (epidemiological, clinical). Most of the serum samples were collected towards the end of the Omicron BA.1 wave and during the Omicron BA.2 wave. By the time the samples were collected for the study, 39% of the Slovenian population had already had a microbiologically confirmed SARS-CoV-2 infection at least once, and approximately 3% of them had been infected more than once (data from the National COVID-19 Database). Fifty-eight percent of the population was completely vaccinated, and 30% of the population had already received at least one additional dose (data from the National Vaccination Register).

### 3.2. Prevalence of Antibodies against SARS-CoV-2

The study included 2899 participants. Anti-S antibodies were detected in 2439 (84.1%) participants, with no difference in relation to gender (cOR 0.88, 95% C.I. 0.69–1.12) (Table 1). Anti-S seroprevalence increased with age, rising from 77.9% for the 0–17 age group to 91.9% for the ≥70 age group. Anti-S seroprevalence was higher among notified COVID-19 cases than among those who were not registered in the National COVID-19 Database (87.6% and 79.9%, respectively). Among those participants who had been partially or completely vaccinated and individuals with at least one additional dose, anti-S prevalence was 98.6%, 96.0%, and 98.9%, respectively. Those who had been vaccinated (regardless of the number of doses received) had a significantly higher anti-S prevalence compared to those who had never been vaccinated (68.4%). The multivariate analysis demonstrated the strongest independent association with the positivity rate and having an additional COVID-19 vaccine dose (aOR 91.7, 95% C.I. 41.4–203.08).

Anti-N antibodies were positive in 1409 (48.6%) participants (Table 1). Seroprevalence decreased with age (from 61.2% in the 0–17 age group to 28% in the ≥70 age group). The seroprevalence of anti-N antibodies among participants without confirmed SARS-CoV-2 infection was less than half that found among those who had been notified as RT-PCR/RAT SARS-CoV-2 positive (28% and 64.9%, respectively, cOR 4.7, 95% C.I. 4.01–5.5). Participants who had never been vaccinated had a significantly higher proportion of anti-N antibodies compared to completely vaccinated persons and those who had received an additional dose (Table 1).

Out of 2899 participants, 71 participants were partially vaccinated and 858 were completely vaccinated with COVID-19 vaccines. Six-hundred forty-six participants received an additional vaccine dose. Out of 71 partially vaccinated participants, the majority (67 participants, 94.4%) received mRNA vaccines. Among 858 completely vaccinated participants, 684 (79.8%) received two doses of mRNA vaccine and 160 (18.6%) received vector-based vaccines. Fourteen (1.6%) participants were vaccinated with one dose of vector-based vaccine followed by an mRNA vaccine as a second dose. There were 646 participants who were additionally vaccinated with mRNA vaccines (none with vector-based vaccines): 467 (71.2%) participants were vaccinated with mRNA vaccines and 176 (27.2%) participants were vaccinated with a vector-based vaccine. Three participants received a combination of vector-based vaccines and mRNA vaccines before the first booster dose.

As expected, previous infection and vaccination status had the most important effect on both anti-S and anti-N positivity status. To further explore this association, we compared different combinations of infections and vaccination statuses, both overall and over time.

Participants who had at least two recorded events stimulating their immune system against SARS-CoV-2 (either infection or vaccination) tested positive for anti-S in more than 90% of cases (between 92.2% and 100%). The highest percentage of anti-S negative serum samples (47%) (Table 2) was observed in participants who were not recorded as having been infected with SARS-CoV-2 in the National COVID-19 Database or had not yet been vaccinated (never infected-never vaccinated). It seems that approximately half of the ‘never infected-never vaccinated’ group had been asymptomatically or symptomatically infected, but had not had their infection confirmed by official testing before serology was performed. In these cases, it is unlikely that anti-S antibodies were present due to vaccination, as all vaccinations against COVID-19 were recorded in the national vaccination database. There is a possibility that a few individuals were vaccinated abroad (and therefore not recorded in the national database), but we are confident that the number of persons vaccinated outside Slovenia was negligible. Approximately one-third (35.5%) of ‘never infected-never vaccinated’ had detectable anti-N antibodies. Of the 479 ‘never infected-never vaccinated’ participants, 146 were anti-S and anti-N positive, 201 were negative for both antibodies, 108 were positive for anti-S and 24 were positive for anti-N without a detectable anti-S antibody.

Participants who had never been vaccinated but had been infected two or more times (infected-never vaccinated) had the highest prevalence of anti-N antibodies (93.7%). Participants who had been infected at least once and vaccinated with at least one dose before the serum sample was taken tested positive for anti-N antibodies in more than half of cases (from 52.4% to 58.9%). The percentage of anti-N positives was the lowest in the ‘never infected-partially vaccinated’ group, but the number of participants was too small to enable a meaningful interpretation of the data. The second lowest anti-N seropositivity was detected among those who were classified as ‘never infected-additional dose’ (18.9%).

Demographic characteristics, history of previous SARS-CoV-2 infection and/or vaccination of double positives (anti-S and anti-N), positives for anti-S only, positives for anti-N only, and double negatives (anti-S and anti-N) are presented in Table 3.

**Figure 2:**
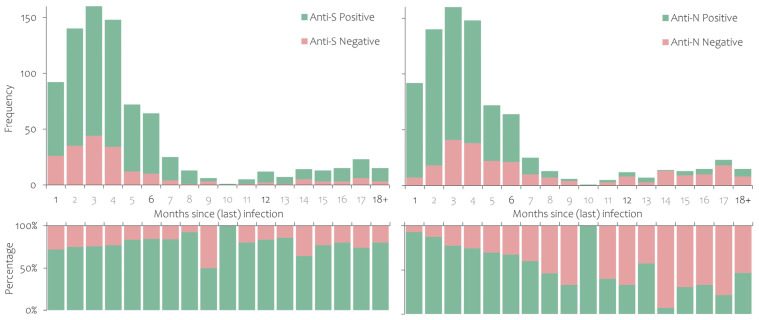
Anti-S and anti-N antibody persistence in study participants who were notified as infected with SARS-CoV-2 but had never been vaccinated with a COVID-19 vaccine.

**Figure 3:**
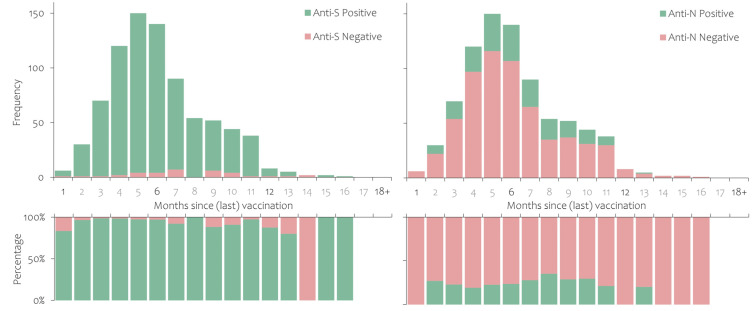
Anti-S and anti-N antibody persistence in study participants who had received at least one dose of a COVID-19 vaccine but without a notified SARS-CoV-2 infection.

**Figure 4:**
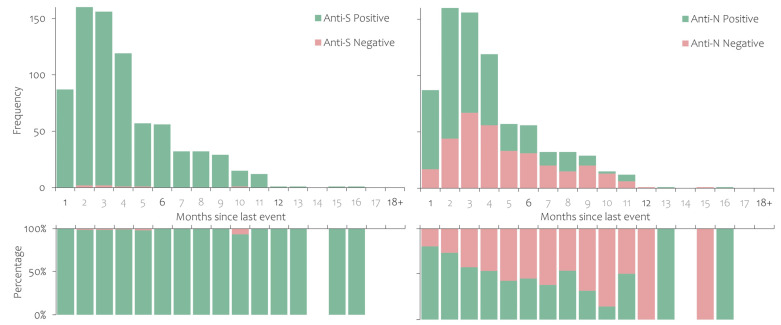
Anti-S and anti-N antibody persistence in study participants who were notified as infected with SARS-CoV-2 and had received at least one dose of a COVID-19 vaccine.

The seroprevalence of anti-S and anti-N according to the time (in months) that had passed since the last vaccination or infection is shown in Figure 2, Figure 3 and Figure 4. Anti-S and anti-N seroprevalence in participants who had recovered from SARS-CoV-2 infection but had never been vaccinated is shown in Figure 2. The proportion of anti-S positives shows no significant time trend, while the proportion of anti-N positives decreased over time in the first six months (92.4% to 67.2%). Participants who had never been infected according to the national register but had been vaccinated with at least one dose of the COVID-19 vaccine had consistently high anti-S seroprevalence, while approximately 20% also had detectable anti-N antibodies (Figure 3).

Finally, among those who were both infected and vaccinated, anti-S antibodies were detected among almost all participants for the entire period under observation (93.3% to 100%). The trend of anti-N antibodies is similar to that seen in those previously infected and never vaccinated, i.e., decreasing over time (from 80.5% to 44.6% in the first six months) (Figure 4).

From the time of serum collection to 14 November 2022, a total of 445 participants (15.3%) tested positive for SARS-CoV-2 (Table 4). Those who tested negative for anti-N and anti-S antibodies were between 40 and 59 years old, and those who did not have a notified previous infection had higher odds of infection. Vaccination status and gender had no significant effects on the risk of infection. When controlling for other variables, middle-aged participants had the highest odds for infection after serum sampling, followed by participants with no anti-S and anti-N antibodies.

## 4. Discussion

This section is not mandatory but the seroprevalence study revealed that by March–June 2022, 84.1% of residual serum samples collected from the Slovenian population contained anti-S antibodies developed after infection or vaccination. The prevalence of anti-N antibodies against the SARS-CoV-2 virus was 48.6%. During the Omicron BA.1 dominance period (the beginning of January to mid-March 2022), around one in every five residents of Slovenia had a confirmed SARS-CoV-2 infection, which explains such a high proportion of seropositives for both types of antibody. The lower prevalence of anti-N antibodies compared to anti-S antibodies was also observed in a longitudinal seroprevalence study of UK HCWs. Serial measurements of anti-N and anti-S IgG SARS-CoV-2 antibodies showed that anti-N antibodies waned within months and anti-S remained stably detected [15]. Another study disclosed that anti-N antibodies frequently became undetectable by 5–7 months [20]. Studies relying on anti-N tests only underestimate the prevalence of earlier infected individuals, as IgG titres might have declined since infection occurred [21].

Routine COVID-19 surveillance underestimates the number of infections–some infections remain asymptomatic and some patients are not tested due to restraints in diagnostic capabilities. Serosurveys helped to understand the true rates of infection and immunity developed after COVID-19 vaccinations were administered. The World Health Organization recognised the importance of seroepidemiological studies in the early phase of the pandemic and recommended a standard approach to follow the development of seropositivity in general and specific populations [22]. The number of seroprevalence studies has expanded over time, showing a global increase in general population seropositivity to the pandemic virus [23,24,25,26,27]. There are a limited number of publications exploring seroprevalence against SARS-CoV-2 after the Omicron BA.1 wave. However, the published studies examining the general population or specific groups, e.g., blood donors or children, uniformly showed a high increase in infection-induced antibodies [28,29,30,31,32,33,34,35,36,37,38]. The wide spread of infections and gradual rise in vaccination coverage in the period covered in this study, although at a slower pace than in 2021, resulted in a high proportion of people with positive SARS-CoV-2 antibodies. In spring 2022, the seroprevalence of anti-S antibodies varied from 81.4% to 97.6%, depending mostly on the timing of serum sample collection, the age of the participants, and vaccination coverage [28,29,33,38,39]. The anti-S seroprevalence found in our study is in accordance with studies published so far. The anti-N seroprevalence increased after the Omicron surge, with the highest positivity rate in the youngest age groups, and this was also found in our convenient sample [35,38]. The prevalence of anti-N antibodies in blood donors from South Africa, the first country hit by the Omicron wave, amounted to nearly 90% in March 2022 [40]. A large national serosurveillance study testing blood donors for anti-N antibodies estimated that 66% of the adult Danish population had been infected by the Omicron variant by March 2022, with at least one-third of infections not captured by RT-PCR testing [36]. Serial cross-sectional studies in British Columbia revealed that infection-induced seroprevalence was less than 15% in early autumn 2021 but increased across Omicron waves to 61% [41]. Despite high vaccination coverage, the emergence of Omicron had an immense impact on infection-related seroprevalence [30].

In our study, seroprevalence did not differ meaningfully when stratified by gender. Some studies with gender-stratified results have found no difference in seroprevalence between males and females [29,32,40,41]. A study from Jamaica reported that females were more likely than males to be nucleocapsid IgG positive, but with similar results to males in the testing of spike RBD IgG antibodies [28]. By contrast, seroprevalence among Canadian blood donors confirmed that anti-N positivity was significantly higher among males [30]. Gender differences in contact rates may result in gender-specific infection rates. A modelling study revealed that there were higher infection risks among women than men at working ages without taking into account infection clusters and super-spreading events [42].

Our study also found that the correlate of infection-induced seroprevalence only, i.e., anti-N antibodies, decreased with age. This age-related finding is in accordance with post-BA.1 seroprevalence studies: children, adolescents, and young adults were the least vaccinated but most infected age subgroups during the Omicron BA.1 wave [28,29,32,34,35,43]. In most countries, the education systems functioned with few restrictions during this period, and children and adolescents were socialising to almost the same extent as before the COVID-19 pandemic. The high transmissibility of the Omicron variant, the relaxation of public health measures, and the low rate of vaccination coverage were thus the main drivers of the high infection rate among children and young people. We can speculate that elderly people were more likely to practise social distancing, wear masks and use other hygiene measures to prevent infection during the Omicron wave as in previous waves. A higher level of COVID-19 risk perception in the elderly was linked to a greater level of engagement in preventive behaviours and even in medical care avoidance in earlier phases of the pandemic [44,45]. Vaccination coverage, as reflected in the high prevalence of anti-S antibodies (without anti-N antibodies) in the ≥60-year group, additionally lowered the chance of at least a more severe infection.

We grouped the participants according to past SARS-CoV-2 infection and vaccination history. Participants who had been vaccinated but never officially recorded as infected with SARS-CoV-2 had the lowest prevalence of anti-N antibodies. Participants who had never been vaccinated had a significantly higher proportion of anti-N antibodies compared to completely vaccinated persons and persons who had received an additional dose. This observation might be explained by more frequent infections in non-vaccinated persons. The most interesting group of participants were those who were never recorded as having been vaccinated or infected. In this group, one-third of the participants had detectable anti-N antibodies and half had detectable anti-S antibodies. If we combine the results from those who had never officially been infected (regardless of their vaccination status), more than a quarter (28.3%, 365/1291) had detectable anti-N antibodies. Some of those participants had probably been infected asymptomatically. We assume that some participants in this group had symptoms and performed a rapid antigen test at home but chose not to do a confirmatory test in a healthcare facility in order to avoid the obligatory seven-day isolation period, or for another reason.

The ‘never infected-never vaccinated’ group included 201 participants with no antibodies detected. It seems that despite two and a half years of the pandemic, some individuals remained both uninfected and unvaccinated, with a high risk of potentially severe infection. For some participants, this finding might also translate into a past infection that was so far back that the detectable antibodies had already fallen under the threshold for the test used.

The analysis of post-serum sampling infections showed that every seventh participant had a positive RT-PCR or RAT for SARS-CoV-2 within half a year or more of serological testing. The risk factors for confirmed post-serum sampling infection were anti-S and anti-N seronegativity and belonging to the 40–59 age group. The high odds of infection in seronegative participants were anticipated. We can speculate that the higher odds of infection in middle-aged people could be a consequence of testing practices and not of higher risk. Middle-aged people generally belong to the working population, and thus if they contract an acute respiratory infection (ARI) have to consult a physician if they wish to stay at home on sick leave. Moreover, during the pandemic, it was recommended that every MAARI (medically attended ARI) be tested for SARS-CoV-2. In contrast, preschool children and students were entitled to a free-of-charge home self-test, most likely did not consult a physician for any minor acute respiratory illnesses, and were not directed to official testing. However, we do not have data regarding age-specific testing practices, so this statement is only speculative.

One limitation of this study is that the use of residual serum samples taken from individuals attending healthcare settings limits the representativeness and therefore the generalisability of the findings. Using residual sera may cause an overrepresentation of more frequent healthcare seekers or people who have better access to healthcare. Policies on sick leave also have an impact on consultation practices. Nevertheless, the results of our study are fairly comparable to those of other publications that took a more systematic approach to sampling.

## 5. Conclusions

Our study showed the high prevalence of anti-S and anti-N antibodies after the Omicron BA.1 wave, with a high burden of infection in the young. The high prevalence of anti-S antibodies (>90%) in seniors reflected the high vaccine coverage and considerable exposure to SARS-CoV-2 in this group. This seroprevalence study, despite its limitations, offers insight into the development of the pandemic in Slovenia and may support informed public health decision making in the future.

## Figures and Tables

**Table 1:** Seroprevalence of anti-S and anti-N antibodies, stratified by gender, age group, previous SARS-CoV-2 infection, and vaccination.

	Participants	Anti-S Positive			Anti-N Positive		
	n	n (%)	Crude OR (95% CI)	Adjusted OR (95% CI)	n (%)	Crude OR (95% CI)	Adjusted OR (95% CI)
Male	672	574 (85.4)	ref	ref	307 (45.7)	ref	Ref
Female	2227	1865 (83.7)	0.88 (0.69–1.12)	1.02 (0.75–1.39)	1102 (49.5)	1.16 (0.98–1.38)	1.10 (0.90–1.35)
0–17	299	233 (77.9)	ref	ref	183 (61.2)	ref	Ref
18–29	748	609 (81.4)	1.24 (0.89–1.73)	0.48 (0.32–0.71)	403 (53.9)	0.74 (0.56–0.97)	0.60 (0.44–0.82)
30–39	757	617 (81.5)	1.25 (0.90–1.74)	0.40 (0.27–0.60)	385 (50.9)	0.66 (0.50–0.86)	0.52 (0.38–0.71)
40–49	306	264 (86.3)	1.78 (1.16–2.72)	0.35 (0.21–0.58)	141 (46.1)	0.54 (0.39–0.75)	0.48 (0.33–0.70)
50–59	327	294 (89.9)	2.52 (1.61–3.97)	0.46 (0.27–0.78)	147 (45.0)	0.52 (0.38–0.71)	0.60 (0.41–0.86)
60–69	251	228 (90.8)	2.81 (1.69–4.67)	0.47 (0.26–0.85)	91 (36.3)	0.36 (0.25–0.51)	0.53 (0.36–0.79)
70+	211	194 (91.9)	3.23 (1.84–5.69)	0.33 (0.16–0.67)	59 (28.0)	0.25 (0.17–0.36)	0.48 (0.31–0.74)
Never infected	1291	1031 (79.9)	ref	ref	365 (28.3)	ref	ref
Infected ≥ 1	1608	1408 (87.6)	1.78 (1.45–2.17)	3.71 (2.92–4.72)	1044 (64.9)	4.70 (4.01–5.50)	4.45 (3.75–5.28)
Never vaccinated	1324	906 (68.4)	ref	ref	775 (58.5)	ref	ref
Partially vaccinated	71	70 (98.6)	32.30 (4.47–233.28)	28.27 (3.89–205.57)	34 (47.9)	0.65 (0.40–1.05)	0.48 (0.29–0.78)
Completely vaccinated	858	824 (96.0)	11.18 (7.79–16.06)	15.91 (10.88–23.26)	410 (47.8)	0.65 (0.55–0.77)	0.73 (0.61–0.89)
Additional dose	646	639 (98.9)	42.12 (19.82–89.50)	91.69 (41.40–203.08)	190 (29.4)	0.30 (0.24–0.36)	0.53 (0.41–0.67)

**Table 2:** Seropositivity of anti-S and anti-N antibodies in participants stratified according to vaccination status and SARS-CoV-2 RT-PCR or RAT positivity recorded in the National COVID-19 Database.

	Anti-S		Anti-N	
	Negative (%)	Positive (%)	Negative (%)	Positive (%)
Never infected and never vaccinated	225 (47.0)	254 (53.0)	309 (64.5)	170 (35.5)
Infected once, never vaccinated	188 (27.4)	499 (72.6)	230 (33.5)	457 (66.5)
Infected ≥ 2, never vaccinated	5 (3.2)	153 (96.8)	10 (6.3)	148 (93.7)
Total for infected, never vaccinated	193 (22.8)	652 (77.2)	240 (28.4)	605 (71.6)
Never infected, partially vaccinated	1 (12.5)	7 (87.5)	7 (87.5)	1 (12.5)
Never infected, completely vaccinated	27 (7.8)	317 (92.2)	237 (68.9)	107 (31.1)
Never infected, additional dose	7 (1.5)	453 (98.5)	373 (81.1)	87 (18.9)
Total for never infected, vaccinated	35 (4.3)	777 (95.7)	617 (76.0)	195 (24.0)
Infected ≥1, partially vaccinated	0 (0.0)	63 (100.0)	30 (47.6)	33 (52.4)
Infected ≥1, completely vaccinated	7 (1.4)	507 (98.6)	211 (41.1)	303 (58.9)
Infected ≥1, additional dose	0 (0.0)	186 (100.0)	83 (44.6)	103 (55.4)
Total for infected, vaccinated	7 (0.9)	756 (99.1)	324 (42.5)	439 (57.5)

**Table 3:** Demographic data, vaccination history, and data on previous infection with SAS-CoV-2 stratified according to anti-S and anti-N antibodies serostatus.

		Anti-S Negative and Anti-N Negative	Anti-S Positive and Anti-N Negative	Anti-S Negative and Anti-N Positive	Anti-S Positive and Anti-N Positive
	n	349	1141	111	1298
Age (years)	median (IQR)	31.0 (25–40)	39 (29–59)	30 (24–39)	32 (26–46)
Gender (male)	n (%)	73 (20.9)	292 (25.6)	25 (22.5)	282 (21.7)
Infected at least once	n (%)	116 (33.2)	448 (39.3)	84 (75.7)	960 (74.0)
Never vaccinated	n (%)	311 (89.1)	238 (20.9)	107 (96.4)	668 (51.5)
mRNA vaccine (1, 2, or 3 doses)	n (%)	22 (6.3)	711 (62.3)	4 (3.6)	481 (37.1)
Vector-based vaccine (1 or 2 doses)	n (%)	14 (4.0)	70 (6.1)	0 (0.0)	80 (6.2)
Combination of different vaccines (2 or 3 doses)	n (%)	2 (0.6)	122 (10.7)	0 (0.0)	69 (5.3)

**Table 4:** Anti-S and anti-N seroprevalence, gender, age groups, registered SARS-CoV-2 infection before sampling, and COVID-19 vaccination status in participants who tested positive for SARS-CoV-2 after sampling.

	Participants	Confirmed Cases	Crude OR (95% CI)	Adjusted OR (95% CI)
	n	n (%)		
Anti-S neg	460	109 (23.7)	Ref.	Ref.
Anti-S pos	2439	336 (13.8)	0.51 (0.40–0.66)	0.66 (0.51–0.85)
Anti-N neg	1490	327 (21.9)	Ref.	Ref.
Anti-N pos	1409	118 (8.4)	0.33 (0.26–0.41)	0.34 (0.27–0.43)
Male	672	92 (13.7)	Ref.	Ref.
Female	2227	353 (15.9)	1.19 (0.93–1.52)	1.21 (0.93–1.59)
0–17	299	33 (11.0)	Ref.	Ref.
18–29	748	112 (15.0)	1.42 (0.94–2.15)	1.25 (0.80–1.94)
30–39	757	114 (15.1)	1.43 (0.95–2.16)	1.22 (0.78–1.89)
40–49	306	60 (19.6)	1.97 (1.24–3.11)	1.73 (1.08–2.79)
50–59	327	63 (19.3)	1.92 (1.22–3.03)	1.71 (1.07–2.75)
60–69	251	36 (14.3)	1.35 (0.81–2.24)	1.11 (0.66–1.87)
70+	211	27 (12.8)	1.18 (0.69–2.03)	0.89 (0.51–1.56)
Never infected	1291	230 (17.8)	Ref.	Excl.
Infected ≥ 1	1608	215 (13.4)	0.71 (0.58–0.87)	Excl.
Never vaccinated	1324	203 (15.3)	Ref.	Excl.
Partially vaccinated	71	15 (21.1)	1.48 (0.82–2.67)	Excl.
Completely vaccinated	858	113 (13.2)	0.84 (0.65–1.07)	Excl.
Vaccinated with additional dose	646	114 (17.6)	1.18 (0.92–1.52)	Excl.

## Data Availability

The anonymised data presented in this study are available upon request from the corresponding author.

## Supplementary Materials

The following supporting information can be downloaded at https://www.mdpi.com/article/10.3390/ijerph20043665/s1, Table S1: Sample Age and Gender Distribution.
