# Supplementary material for: Seroprevalence of Anti-SARS-CoV-2 Antibodies Following the Omicron BA.1 Wave
Source: Int J Environ Res Public Health. 2023 Feb 18;20(4):3665. doi: 10.3390/ijerph20043665 (PMC9959557; doi:10.3390/ijerph20043665)
Supplement: Supplementary file 1 [file ijerph-20-03665-s001.zip › ijerph-2204005-supplementary.pdf]

Table S1. Sample age and gender distribution.

	**Female (%)**	**Male (%)**
0 - 17	137 (6.2)	162 (24.1)
18 - 29	686 (30.8)	62 (9.2)
30 - 39	682 (30.6)	75 (11.2)
40 - 49	208 (9.3)	98 (14.6)
50 - 59	223 (10)	104 (15.5)
60 - 69	149 (6.7)	102 (15.2)
70 - 79	107 (4.8)	58 (8.6)
80+	35 (1.6)	11 (1.6)
Total	2227 (100)	672 (100)
